# Postoperative Femoral Neuropathy After Oncological Pelvic Surgery: A Case Report and Call for Action

**DOI:** 10.7759/cureus.116632

**Published:** 2026-09-21

**Authors:** André Pereira, Carolina Carvalho, Mariana G Xavier, André O Cruz, Inês Jorge

**Affiliations:** 1 Physical Medicine and Rehabilitation, Unidade Local de Saúde do Alto Minho, Viana do Castelo, PRT

**Keywords:** case report, femoral neuropathy, iatrogenic nerve injury, oncological surgery, pelvic surgery, rehabilitation

## Abstract

Femoral neuropathy is an uncommon but potentially disabling complication that is well described after gynecologic and colorectal surgery, yet remains poorly characterized following extensive pelvic oncological procedures, despite comparable or greater cumulative risk. We describe a 62-year-old man with type 2 diabetes who developed left-sided femoral neuropathy after open cystoprostatectomy, sigmoid colectomy, cutaneous ureterostomy, and pelvic lymphadenectomy for urachal adenocarcinoma. Postoperatively, he presented with hip flexion and knee extension weakness, anterior and medial thigh/leg hypoesthesia, and an absent patellar reflex. Neurophysiological studies supported the diagnosis of femoral neuropathy and identified an underlying chronic axonal sensorimotor polyneuropathy. A multifactorial injury combining compression, traction, and ischemia related to surgical positioning and exposure, retractor use, and intraoperative hypotension was presumed. Despite structured rehabilitation, partial motor and sensory deficits persisted at the two-year follow-up. This case highlights the importance of awareness and prevention of iatrogenic femoral neuropathy, particularly in high-risk procedures and patients with comorbidities. We present a summary of practical preventive measures and a structured diagnostic and management pathway to support earlier recognition and more consistent care, while also highlighting recurring gaps in perioperative documentation and preoperative neurological assessment that limit diagnostic certainty.

## Introduction

The femoral nerve originates from the posterior divisions of the ventral rami of the L2-L4 spinal nerve roots and descends through the psoas major muscle, emerging from its lateral border and continuing between the psoas and iliacus muscles before passing beneath the inguinal ligament into the thigh. Although its relatively deep anatomical course provides protection from external injury, its intrapelvic trajectory makes it vulnerable during pelvic and retroperitoneal procedures, particularly to compression, traction, and ischemia [1].

Postoperative femoral neuropathy is an uncommon but potentially disabling complication. Its true incidence following pelvic surgery remains uncertain, with available estimates deriving predominantly from gynecological and gynecologic-oncological surgical populations. Femoral neuropathy has been reported in 0.25% of patients undergoing major pelvic surgery [2], while a more recent series of gynecologic-oncologic procedures reported an incidence of 1.5% [3]. Differences in surgical procedures, patient populations, diagnostic criteria, and potential underrecognition and underreporting may account for the variability in reported rates.

In the perioperative setting, femoral nerve injury may result from several mechanisms. These include direct compression, particularly from pelvic retractors or postoperative hematoma; excessive traction associated with surgical positioning; ischemia secondary to prolonged pressure or vascular compromise; and, less commonly, direct nerve injury during surgical dissection. Prolonged hip flexion, abduction, and external rotation during lithotomy positioning may increase nerve tension or compression. These mechanisms are particularly relevant during prolonged pelvic and retroperitoneal procedures and may coexist in a multifactorial injury [4-7].

The femoral nerve provides motor innervation to the iliacus and quadriceps muscles and sensory innervation to the anterior thigh and, through the saphenous nerve, the medial leg. Femoral neuropathy therefore typically presents with weakness of knee extension, resulting in knee instability and impaired ambulation, together with sensory disturbance over the anterior thigh and medial leg and a diminished or absent patellar reflex. More proximal involvement may additionally impair hip flexion. The main differential diagnoses include L2-L4 radiculopathy and lumbar plexopathy, which may produce overlapping motor and sensory deficits but typically involve additional muscles outside the femoral nerve distribution. A careful neurological examination, complemented by imaging and electrodiagnostic studies when appropriate, is therefore important for lesion localization and exclusion of alternative diagnoses [1].

We report a case of postoperative femoral neuropathy following major pelvic oncological surgery and discuss its clinical and anatomical context, highlighting perioperative risk factors, potential mechanisms of nerve injury, diagnostic considerations, rehabilitation and functional outcomes, with particular emphasis on prevention and implications for future surgical care.

## Case presentation

We describe a 62-year-old man with a history of hypertension, dyslipidemia, type 2 diabetes, a left proximal femur fracture previously treated with intramedullary nailing, and a former smoker. His body mass index was 22.4 kg/m², and he had no history of prior chemotherapy exposure. He was ambulating independently without walking aids prior to admission, although no formal preoperative neurological examination had been performed.

He underwent an open cystoprostatectomy, sigmoid colectomy, cutaneous ureterostomy, and pelvic lymphadenectomy for locally invasive urachal adenocarcinoma, under combined spinal and general anesthesia. The procedure lasted approximately four hours and 30 minutes (skin incision to closure), performed in the lithotomy position for a total of nearly five hours. Central venous access was obtained via the right internal jugular vein and arterial monitoring via the left radial artery (no femoral vascular access). Pelvic retractors were used during the procedure (the specific type and duration were not documented). The intraoperative course included a period of relative hypotension, with mean arterial pressure ranging between 60 and 65 mmHg for approximately 20 minutes, managed with vasopressor support (ephedrine and phenylephrine), together with an estimated blood loss of 700 mL.

On the second postoperative day, during the first attempt at mobilization out of bed, the patient was unable to stand and reported difficulty with knee extension and hip flexion, together with sensory loss in the left leg. Whether these deficits were already present earlier in the postoperative course could not be determined, as no neurological assessment had been performed until then. Neurological examination revealed grade 3 weakness of hip flexion and grade 1 weakness of knee extension (according to the Medical Research Council Scale for Muscle Strength - MRC [8]), hypoesthesia of the anterior and medial aspects of the thigh and leg, and absence of the patellar reflex. Hip abduction and adduction, knee flexion, and foot dorsiflexion were preserved (MRC grade 5), and the Achilles reflexes were present and symmetric. Sensation over the proximal medial thigh was preserved. Overall, these findings were consistent with femoral neuropathy.

Although compatibility with the patient's orthopedic femoral implant had not been previously established, a lumbar MRI under an adapted safety protocol was urgently performed to exclude a hemorrhagic complication related to spinal anesthesia as a differential diagnosis, which was ruled out (Figure 1A, 1B). The study also showed no evidence of foraminal or nerve root compromise (Figure 1C-1F). By contrast, abdominal/pelvic MRI was withheld due to this same uncertainty, given the proximity of the orthopedic hardware to the pelvic region and a judged risk exceeding the expected diagnostic benefit. CT imaging was performed instead and showed no evidence of hematoma, collection, residual tumor, or other local surgical complication along the expected course of the femoral nerve that could account for the neuropathy (Figure 2).

**Figure 1:**
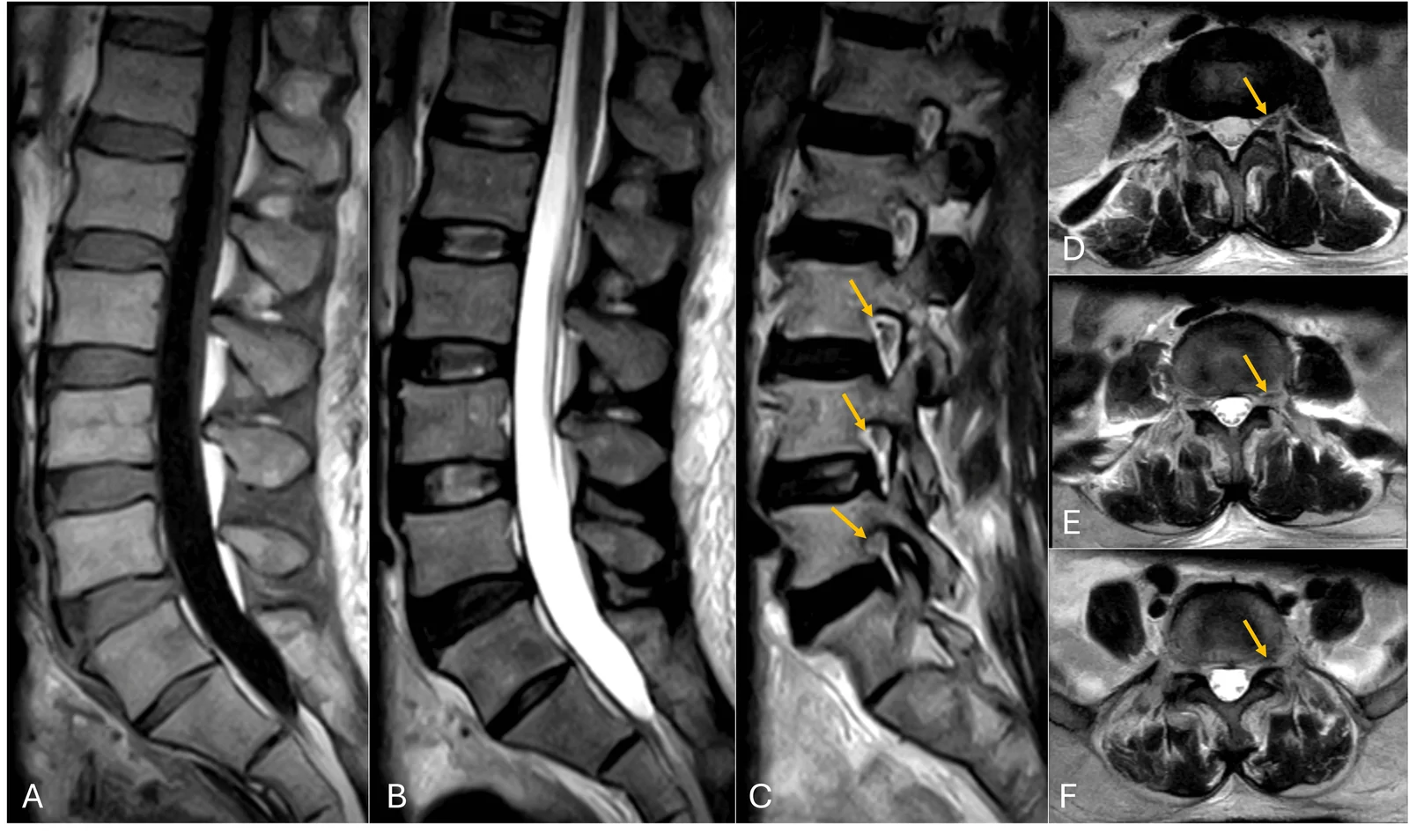
Lumbar spine MRI. (A) Sagittal T1-weighted image, (B) sagittal T2-weighted midline image, (C) sagittal T2-weighted paramedian image, and (D–F) axial T2-weighted images at the L2–L3, L3–L4, and L4–L5 levels showing preserved spinal canal caliber with no evidence of epidural hematoma, abscess, cerebrospinal fluid collection, or nerve root compression (yellow arrows).

**Figure 2:**
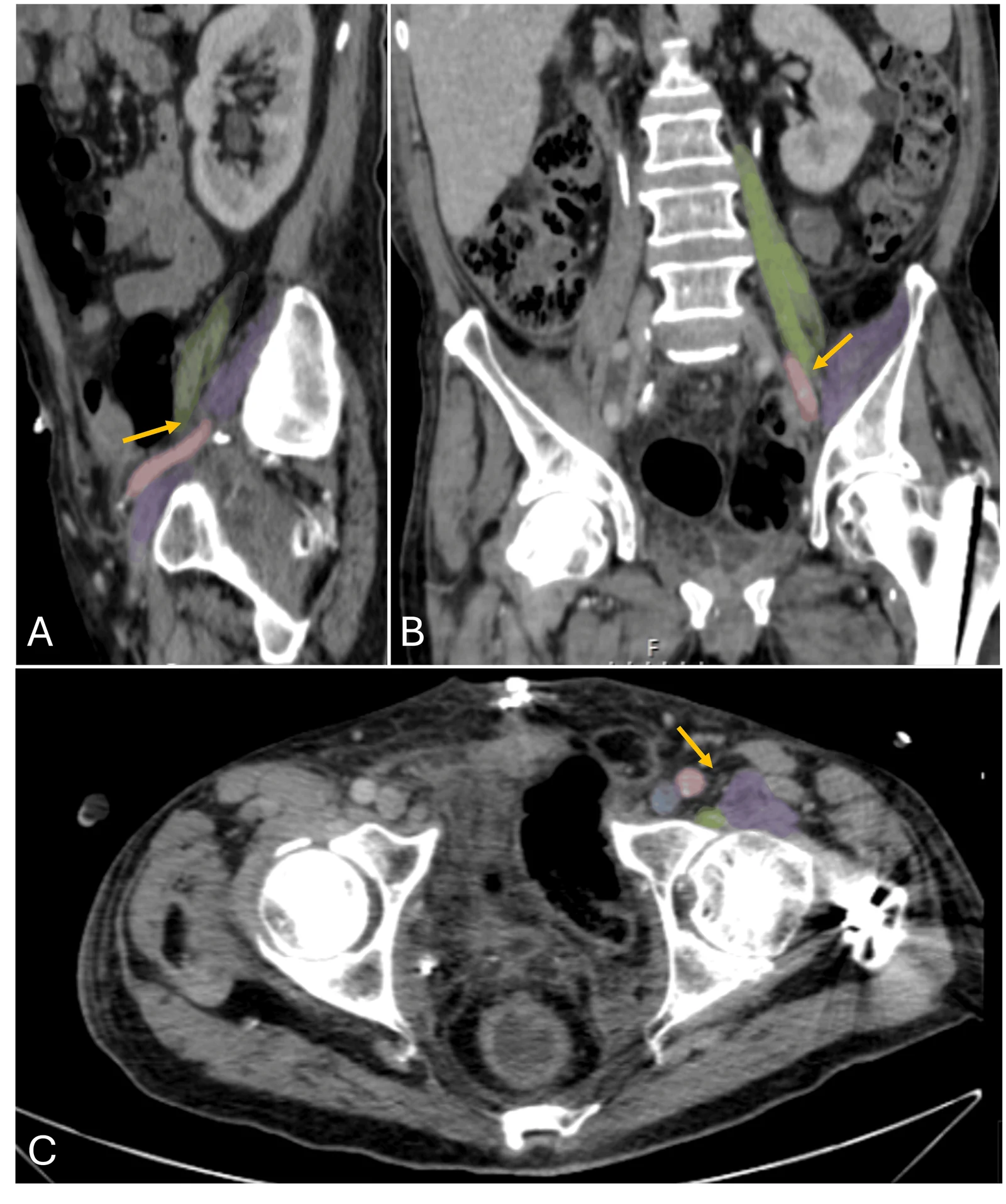
CT images of the abdomen and pelvis. Sagittal (A), coronal (B) and axial (C) views. The *iliacus* muscle is highlighted in purple, the *psoas major* muscle in green, the femoral artery in red, and the femoral vein in blue. No local surgical complications or residual tumor are observed along the expected course of the femoral nerve (yellow arrows).

Nerve conduction studies, performed six weeks after surgery, showed an absent/non-recordable left saphenous sensory response, with a reduced-amplitude response on the right side (Table 1). Motor and sensory conduction studies of the median and ulnar nerves were normal bilaterally, while peroneal motor, sural sensory, and superficial peroneal sensory amplitudes were bilaterally reduced with relatively preserved conduction velocities, a length-dependent pattern consistent with an underlying chronic axonal sensorimotor polyneuropathy (Table 1). Femoral motor nerve conduction studies were not performed. Needle electromyography of the iliacus and vastus lateralis and medialis muscles showed active denervation (fibrillation potentials and positive sharp waves) together with neurogenic changes in motor unit morphology (increased amplitude, duration, and polyphasia) and markedly reduced voluntary recruitment, while the adductor longus was normal bilaterally - findings supporting the clinical suspicion of femoral neuropathy. Milder chronic neurogenic changes, with normal to mildly reduced recruitment, were also noted in more distal muscles (tibialis anterior, gastrocnemius, peroneus longus) bilaterally (Table 2).

**Table 1:** Motor and sensory nerve conduction studies, performed six weeks after surgery. APB: abductor pollicis brevis; ADM: abductor digiti minimi; AHB: abductor hallucis brevis; EDB: extensor digitorum brevis; L: left; R: right Motor amplitudes are reported in mV; sensory amplitudes are reported in μV.

Nerve and site	Recording Site	Latency (ms)	Amplitude	Conduction velocity (m/s)
Motor nerve conduction studies				
R medianus	APB	4.21	6.6	54.7
L medianus	APB	4.33	7.1	50.7
R ulnaris	ADM	3.03	6.2	50.9
L ulnaris	ADM	3.15	7.3	51.8
R tibialis	AHB	4.56	5.5	44.7
L tibialis	AHB	4.86	5.9	45.7
R peroneus	EDB	4.12	1.7	47.5
L peroneus	EDB	3.81	1.7	45.1
Sensory nerve conduction studies				
R medianus	Digit 3	3.21	18.5	53.1
L medianus	Digit 3	3.35	17.9	51.6
R ulnaris	Digit 5	3.01	15.2	52.5
L ulnaris	Digit 5	3.01	16.8	49.9
R suralis	Posterior ankle	3.77	3.3	42.4
L suralis	Posterior ankle	3.72	3.3	43.0
R peroneus superficialis	Lateral ankle	3.60	3.7	44.4
L peroneus superficialis	Lateral ankle	3.65	3.8	43.7
R saphenus	Medial ankle	3.78	2.1	40.1
L saphenus	Medial ankle	Absent/non-recordable		

**Table 2:** Needle electromyography (EMG) findings by muscle. Amp: amplitude; Dur: duration; Fasc: fasciculation potentials; Fib: fibrillation potentials; L: left; MUAP: motor unit action potential; N: normal; PPP: polyphasic potentials; PSW: positive sharp waves; R: right

Muscle	Spontaneous activity			MUAP			Recruitment pattern
	Fib	PSW	Fasc	Amp	Dur	PPP	
L iliacus	0	4/10	0	+	+	+	Markedly reduced
L vastus lateralis	0	5/10	0	+	+	+	Markedly reduced
L vastus medialis	0	6/10	0	+	+	+	Markedly reduced
L adductor longus	0	0	0	N	N	N	N
L tibialis anterior	0	0	0	+	+	+	N
L gastrocnemius caput mediale	0	0	0	+	+	+	Mildly reduced
L peroneus longus	0	0	0	+	+	+	Mildly reduced
R vastus medialis	0	0	0	N	N	N	N
R adductor longus	0	0	0	N	N	N	N
R gastrocnemius caput mediale	0	0	0	+	+	+	Mildly reduced
R tibialis anterior	0	0	0	+	+	+	N

A multifactorial perioperative injury to the femoral nerve was considered most likely. Potential contributing mechanisms included compression or traction associated with prolonged lithotomy positioning, retractor use, and surgical manipulation during pelvic lymphadenectomy, given the anatomical proximity of the operative field to the femoral nerve. Transient intraoperative hypotension may also have contributed through neural ischemia. However, in the absence of direct intraoperative evidence of nerve injury, a single causal mechanism could not be established. Oral vitamin B-complex supplementation (B1 100 mg, B12 200 mcg) was initiated twice daily, and a structured rehabilitation program began on the day following identification of the deficits to support nerve recovery and improve mobility and performance in activities of daily living.

Daily inpatient rehabilitation was provided during the approximately three-week hospital stay, comprising verticalization training with a wheeled walker and a hinged knee orthosis locked in extension (Figure 3) - necessary to stabilize the knee during stance and prevent posterior knee soft tissue strain - active-assisted mobilization, neuromuscular recruitment facilitation techniques targeting the affected muscle groups, including neuromuscular electrical stimulation (charge-balanced biphasic rectangular pulses with 35-50 Hz frequency and 200-400 μs duration), sensory re-education using varied textures and materials, and gait and balance training progressively adapted to functional recovery. Strengthening exercises targeting the hip flexors and knee extensors were progressively introduced as motor recovery allowed.

**Figure 3:**
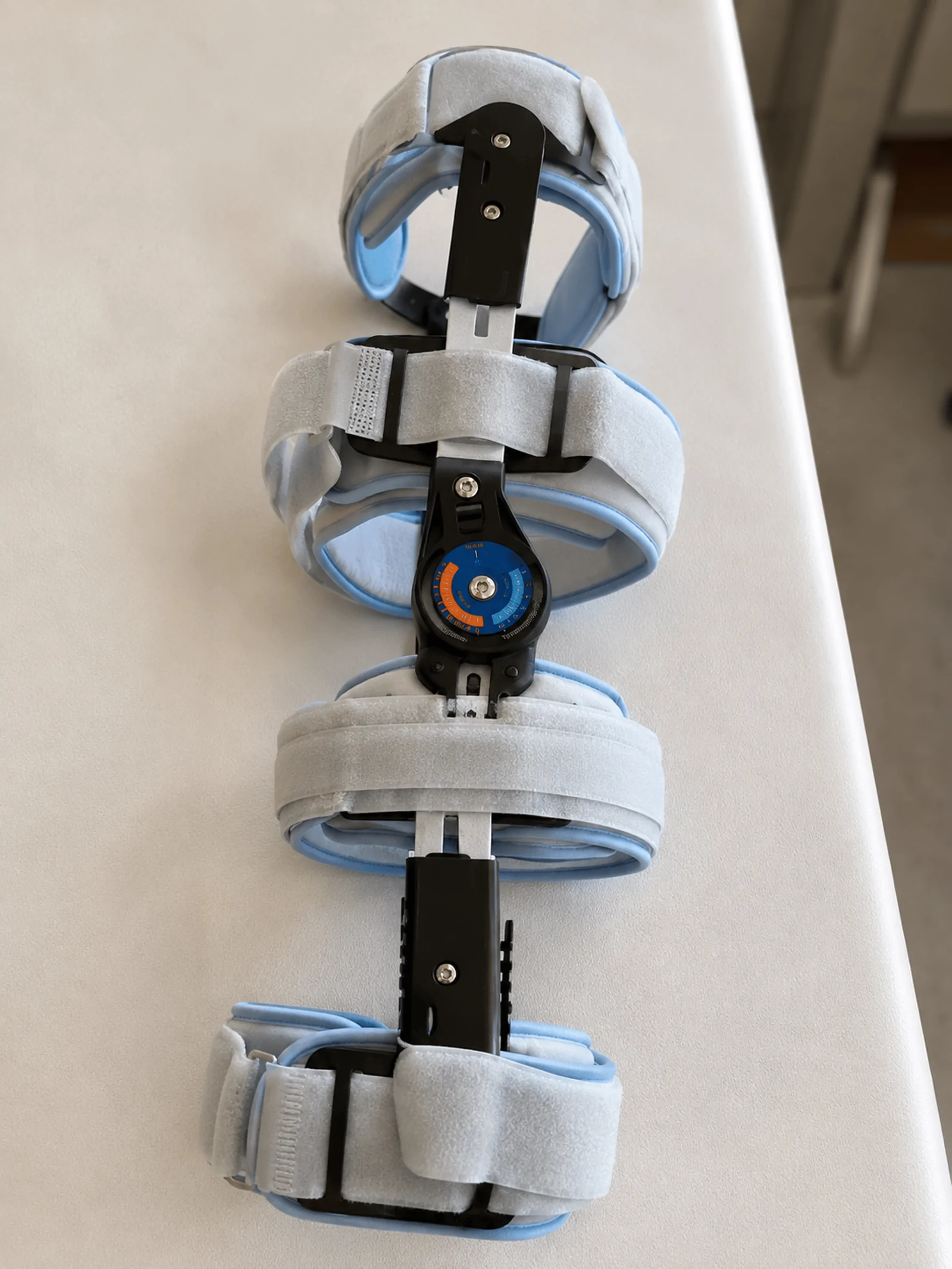
Postoperative knee orthosis used during rehabilitation. Adjustable range-of-motion hinged knee orthosis used during the inpatient and outpatient rehabilitation program, initially locked in extension to restrict knee flexion and protect the posterior knee soft tissues, and progressively unlocked to permit controlled range of motion as motor recovery advanced.

Following discharge, outpatient rehabilitation continued daily for approximately three months, then two to three times weekly through the first year, after which the patient transitioned to autonomous exercise in a gym setting. Reassessments were performed at three, six, 12, 18, and 24 months. At three months, the patient showed MRC grade 3 hip flexion and MRC grade 2 knee extension, with subjective improvement in sensory loss, and was ambulating independently with forearm crutches and the knee orthosis. At six months, strength had progressed to MRC grade 3 in both hip flexion and knee extension, improving active knee control. The orthosis was subsequently weaned and retained only for anticipated longer-distance walking, while the patient transitioned to a single forearm crutch. By 12 months, the patient had reached MRC grade 4 strength in hip flexion and knee extension and reported continued subjective sensory improvement. Although difficulty climbing and descending stairs persisted, requiring handrail support, he could walk without walking aids or orthosis. The patient described a slowing of motor and functional recovery from around 10 months onward. Repeat neurophysiological studies, performed at 12 months, showed resolution of active denervation (positive sharp waves and fibrillation potentials absent), with persistent reinnervation changes (increased motor unit amplitude, duration and polyphasia) and mild to moderately reduced recruitment of iliacus, vastus lateralis and vastus medialis, consistent with a partially reinnervated state without evidence of ongoing axonal loss. Physical examination findings at 18 months were unchanged relative to those at 12 months.

At the two-year follow-up, the patient maintained MRC grade 4 weakness in hip flexion and knee extension, a persistent 2 cm asymmetry in mid-thigh circumference compared with the contralateral side, and mild residual sensory loss. He was able to walk long distances with no clinically evident gait abnormality, though he continued to require a handrail when using stairs.

## Discussion

Although often underrecognized and underreported, postoperative femoral neuropathy is a well-established complication of gynecologic, colorectal, and orthopedic surgery, with much of the available evidence on its mechanism, prevention, and management derived from these settings [2,6,7,9-11]. This case occurred in a comparatively underrepresented context: extensive combined oncological pelvic surgery for a rare malignancy. Sigmoid colectomy features among the colorectal procedures associated with femoral nerve injury [11], and its combination with pelvic lymphadenectomy, prolonged lithotomy positioning, and retractor use illustrates how overlapping surgical fields and exposures may compound cumulative perioperative nerve stress. This combination of risk factors, arising from extensive oncological pelvic surgery rather than a single well-characterized operation, has received comparatively little specific attention in the literature.

A multifactorial mechanism is most consistent with this presentation, and no single causal mechanism can be established. Prolonged lithotomy positioning can expose the femoral nerve to traction and compression, particularly with excessive hip flexion, abduction, or external rotation, with risk increasing with prolonged duration [4,6,10,11]. Pelvic retractors may additionally compress the nerve directly or against the psoas and pelvic sidewall [10], while dissection and retraction during pelvic lymphadenectomy may further increase mechanical stress because of the proximity of the operative field to the nerve's intrapelvic course. An ischemic component related to the documented period of intraoperative hypotension may have acted in combination, as peripheral nerves have limited capacity to autoregulate endoneurial blood flow under sustained compression or traction, and systemic hypoperfusion during such periods can lower the threshold for ischemic injury even at pressures and durations that might otherwise be tolerated [12,13]. In addition, left-sided vulnerability has been attributed to comparatively limited collateral arterial supply and to greater lateral iliopsoas retraction during rectosigmoid exposure [10,11]. Conversely, femoral vascular catheterization, another recognized iatrogenic mechanism, was not performed in this case [4,6]. Importantly, the specific type and duration of retractor use were not documented in the operative or anesthesia records, precluding a more definitive mechanistic attribution [6,7,9,14]. Published series have shown that the use of self-retaining retractors can increase the incidence of postoperative femoral neuropathy by more than tenfold [15]. Given the plausible mechanistic relevance of retractor use, the absence of detailed documentation in this case illustrates a recurring limitation in perioperative records and reinforces the value of routine, structured intraoperative recording of retractor type, positioning, and duration, particularly during prolonged, high-risk procedures. Such documentation represents a simple and readily implementable measure to support both preventive practice and the subsequent evaluation of postoperative neurological complications. More broadly, integrating preventive strategies into oncological surgical protocols may help reduce postoperative neurological morbidity. Table 3 summarizes practical, low-cost measures addressing retractor placement, patient positioning, systemic risk-factor optimization, and intraoperative documentation [6,7,9-11,15].

**Table 3:** Practical preventive measures in pelvic surgery.

Category	Measure
Positioning	Avoid excessive hip flexion, abduction, and external rotation during lithotomy positioning. Limit the duration of lithotomy positioning whenever feasible. Periodically reassess patient positioning during prolonged procedures and temporarily modify positioning when feasible.
Retractor	Consider manual retraction over self-retaining systems. Confirm a visible or palpable clear space between the retractor blade and the psoas muscle before securing it. Use the shortest retractor blade sufficient for adequate exposure. Cushion the blades with padding material. Periodically reinspect retractor position throughout the procedure and temporarily release pressure when feasible.
Systemic factors	Maintain appropriate perioperative glycemic control. Monitor for and promptly correct intraoperative hypotension. Encourage preoperative smoking cessation and reduction of alcohol use, when applicable. Identify and optimize other relevant pre-existing metabolic or nutritional risk factors when clinically indicated.
Intraoperative documentation	Record retractor type, positioning, duration, and any temporary pressure release during the procedure. Record total duration in lithotomy position and any temporary positioning adjustments made. Record the type and site of vascular access obtained. Record the timing, magnitude, and duration of any intraoperative hypotensive episodes.

Several patient-related factors likely contributed to susceptibility to nerve injury and the subsequent prolonged and incomplete recovery. The patient's age plausibly contributed to the comparatively slow and incomplete recovery observed, as advancing age is among the most consistent predictors of functional outcome and is associated with reduced peripheral nerve regenerative capacity [16,17].

More importantly, type 2 diabetes and the electrodiagnostically identified chronic axonal sensorimotor polyneuropathy may have increased vulnerability to superimposed perioperative injury, consistent with a “double-crush”-type mechanism [12,13]. Diabetes has also been associated with delayed recovery from femoral and other perioperative neuropathies [5,6,10]. Smoking has been associated with impaired peripheral nerve recovery through adverse vascular, cellular, and immunomodulatory effects [18,19], supporting smoking cessation as part of preoperative risk-factor optimization in active smokers. However, as this patient had stopped smoking before surgery, the contribution of previous tobacco exposure to his susceptibility or recovery cannot be established.

The electrophysiological findings warrant careful interpretation regarding lesion localization. Needle EMG demonstrated active denervation and neurogenic MUAP changes in the iliacus and quadriceps femoris, while the adductor longus was normal. Because the branch supplying the iliacus arises from the femoral nerve proximal to the inguinal ligament, its involvement favors an intrapelvic lesion, lending further support to the intrapelvic mechanisms discussed above. Preservation of the obturator-innervated adductor longus argues against a more diffuse lumbar plexus process [1]. However, neither psoas major (innervated directly by branches of the lumbar plexus) nor lumbar paraspinal muscles (innervated segmentally by the posterior primary rami of the corresponding nerve roots) were examined, representing a genuine limitation precluding formal electrophysiological exclusion of a more proximal lesion [13]. Femoral motor nerve conduction studies (CMAP) were similarly not performed, limiting quantification of axonal loss and prognostic assessment, since the degree of CMAP amplitude reduction across the lesion correlates with expected recovery [13].

The mild neurogenic changes in the distal muscles are more plausibly explained by the concurrent length-dependent axonal sensorimotor polyneuropathy, whereas the disproportionate left-right asymmetry in saphenous sensory conduction, exceeding what the background polyneuropathy would predict, supports an additional localized femoral nerve lesion via its saphenous branch.

Clinical and imaging findings provided additional support for a femoral nerve localization. Preserved hip adduction and foot dorsiflexion argue against significant obturator nerve or L4 root involvement and against a more diffuse plexus process, while lumbar MRI demonstrated no nerve root compromise. Thus, although a more proximal lesion cannot be formally excluded, the convergence of the clinical, imaging, and electrophysiological findings most strongly supports femoral neuropathy.

The absence of a formal preoperative neurological examination remains an important limitation. This is particularly relevant given the previous ipsilateral proximal femur fracture and intramedullary fixation, which could potentially have resulted in pre-existing neurogenic or myopathic abnormalities. Independent ambulation without walking aids before admission provides indirect reassurance against a clinically significant deficit, but this functional observation is not equivalent to a structured neurological examination and cannot exclude subclinical involvement. Moreover, because the first documented neurological examination occurred on postoperative day 2, the exact onset of the deficit cannot be established. These limitations support incorporating a brief targeted neurological baseline examination into the routine preoperative assessment of patients undergoing prolonged or high-risk pelvic procedures, particularly when previous surgery, trauma, or neurological comorbidity may affect the nerve territories at risk. Such assessment may prevent the erroneous attribution of pre-existing deficits to a perioperative event and establish a reliable reference against which postoperative findings can be compared [12,13].

Postoperative femoral neuropathy benefits from a structured diagnostic and management approach (Figure 4). Time-sensitive central or neuraxial causes should be promptly excluded when clinically suspected. In severe, progressive, or atypical presentations, particularly following pelvic or abdominal surgery, early imaging should also be considered to exclude a potentially reversible local compressive complication, even in the absence of specific signs or symptoms. Localization-focused examination and early functional support should proceed concurrently, without awaiting full diagnostic certainty [12]. Electrodiagnostic assessment is best planned according to the timing and severity of symptoms (typically over the first one to three weeks after injury), as studies performed too early may underestimate axonal injury because denervation may not be fully evident until Wallerian degeneration has progressed. Repeat testing, if feasible, can add prognostic value by demonstrating either early reinnervation or persistent denervation [12,13]. In this case, the six-week study demonstrated active denervation, while the 12-month reassessment documented resolution of active denervation with persistent reinnervation changes and reduced recruitment, consistent with partial reinnervation.

**Figure 4:**
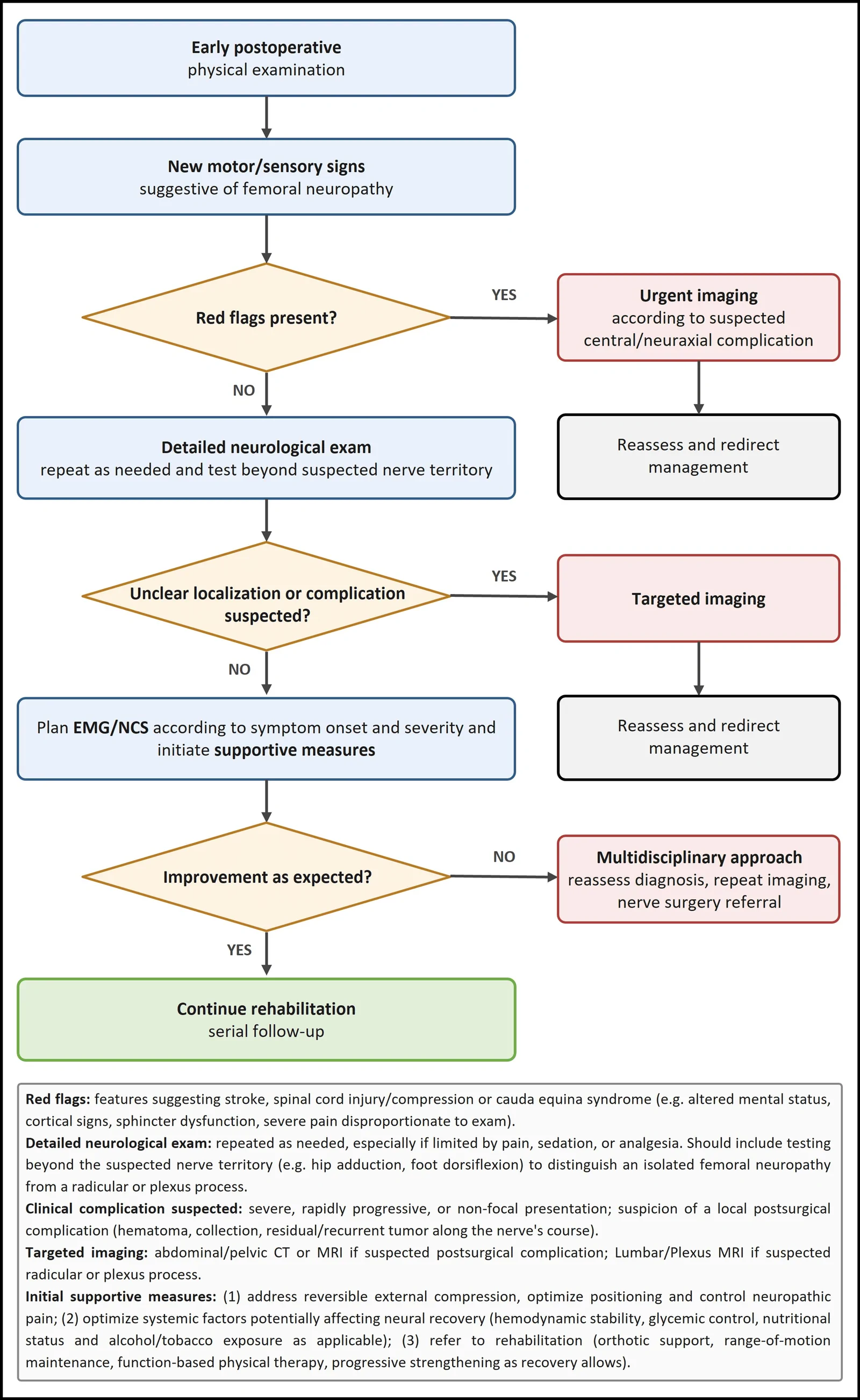
Stepwise approach to suspected postoperative femoral neuropathy: diagnosis and management.

Rehabilitation should begin early and proceed concurrently with the diagnostic workup, prioritizing maintenance of range of motion, safe mobility, orthotic support when quadriceps weakness compromises stability, and progressive strengthening as motor recovery allows [10,12,13]. This approach was implemented through the patient's intensive inpatient and subsequent outpatient rehabilitation program and is consistent with the general principle that early structured rehabilitation improves mobility, reduces complication rates, and promotes functional independence in patients with peripheral nerve injuries [20].

The expected prognosis of postoperative femoral neuropathy varies considerably with injury mechanism and severity. Near-complete or complete recovery has been reported in approximately 48% of postoperative femoral neuropathies, with mean recovery times of up to 14.8 months [5], while predominantly compressive injuries in gynecologic and colorectal series have shown recovery rates of up to 94% [10,11]. Greater axonal loss or an ischemic component may result in a more prolonged course, with recovery continuing for up to 24 to 36 months [13]. In this patient, motor recovery, consistent with a lesion at the more severe end of this spectrum, progressed predominantly during the first year and subsequently slowed, paralleling the electrophysiological evidence of partial reinnervation. Persistent quadriceps atrophy and sensory loss at two years suggest that substantial residual deficits may persist, although further gradual improvement cannot be excluded given reports of continued reinnervation beyond this interval [13].

## Conclusions

This case underscores the need for heightened awareness among surgeons regarding the clinical implications of femoral neuropathy. Although this complication is well-documented in obstetric and gynecologic literature, where the lithotomy position is commonly used and femoral neuropathy is a recognized iatrogenic nerve injury, this concern should be expanded to other surgical specialties, particularly in the field of oncological surgery. Patients in these settings are often exposed to similar or even greater cumulative risk factors.

Two correctable gaps identified in this case merit particular attention: the absence of documented retractor parameters and of a preoperative neurological baseline, both addressable through structured intraoperative documentation and routine baseline neurological screening before high-risk pelvic procedures. Wider adoption of a standardized diagnostic and management algorithm, such as the one proposed here, may further support consistent recognition and care across surgical teams and specialties.
